# Catalyst-free synthesis of low-temperature thermally actuated shape memory polyurethanes with modified biobased plasticizers

**DOI:** 10.1039/d2ra06862a

**Published:** 2022-12-21

**Authors:** Basharat Ali, Muhammad Atif, Muhammad Perviaz, Adnan Irshad, Muhammad Abdullah, Muhammad Ahmad Mobeen

**Affiliations:** Chemistry Department, University of Education Lahore Vehari Campus, Officers' Colony Vehari-61100 Punjab Pakistan chemistatif@yahoo.com muhammad.atif@ue.edu.pk +92-3024757979; Department of Basic & Applied Chemistry, Faculty of Science & Technology, University of Central Punjab Lahore Pakistan

## Abstract

Recent years have seen research into developing specific application-based materials with particular components. Bio-based polyurethanes (PUs) with self-tightening effect through shape recovery at low temperature have been designed from sesame oil-based plasticizer (HSSO). Without using a catalyst, the produced plasticizer was used to create PU samples. In contrast, orcein-based PU has been created both with and without HSSO. The prepared samples have been analyzed through instrumental as well as chemical analyses for surface chemistry, thermal stability and morphology. The gel content and water absorption capacity of HSSO based PU samples has been observed to be 99.27% and 14.94%, respectively. Shape memory study of the PU samples revealed that HSSO-based PU showed fast shape recovery at 60 °C with shape recovery rate (*R*_r_) and shape fixing rate (*R*_f_) of 94.44% and 5%, respectively, in 150 seconds, whereas at 36 °C the sample showed 85% *R*_r_ in 15 minutes with 93.1196 N force and 52.78% *R*_r_ without force. Low-temperature thermal actuation and high water uptake highlight the prepared samples as suitable candidates for self-tightening structures in textile and biomedical fields.

## Abstract 2

Recent years have seen research into developing specific application-based materials with particular components.

## Graphical abstract

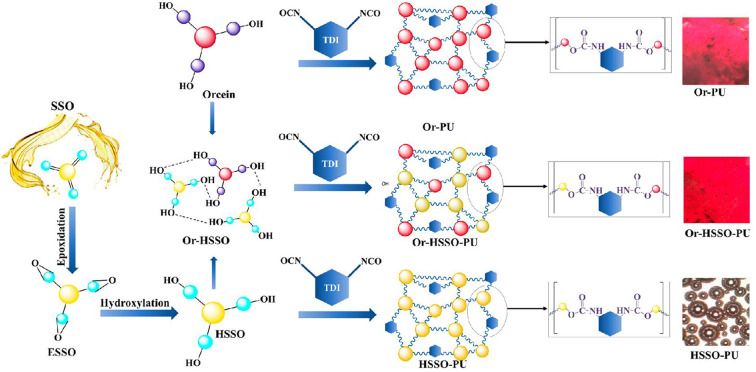

## 1 Introduction

Stimulus-responsive restoration of a polymeric material to its original shape after specific deformation has opened a new dimension in material applications.^1^ These stimuli include, but are not limited to, changes in temperature,^2^ light,^3^ electricity,^4^ pH,^5^ and other similar variables. Biomedical devices such as cardiovascular stents,^6^ sutures,^7^ drug-eluting stents^8^ and clot removal devices,^9^ and tissue engineering,^10^ have made extensive use of SMPs due to their biocompatibility,^11^ biodegradability,^11^ and human body temperature shape recovery.^12–14^ With their tunable transition temperatures for shape recovery and high levels of biocompatibility, shape memory polyurethanes (SMPUs) have shown great promise as responsive materials for use in biomedical devices inside the human body,^12–14^ accompanied by coating material, packing materials and smart textile.^15–17^

However, environmental concerns and exhausted petroleum reserves have shifted the focus to SMPUs with biobased origin rather than petrochemical origin.^18^ Currently, vegetable oils like soyabean oil,^19^ castor oil,^20^ sunflower oil,^21^ jatropha oil^22^ and palm kernel oil,^23^ are the most common source for bio-based SMPUs (bio-SMPUs), but slow curing,^24^ catalytic constraint^25,26^ and reduced elastic strength^27–29^ has limited their applications in comparison to petrochemical based SMPUs. This is because the micro-phase separation of PU is negatively impacted by the lengthy dangling chains present in vegetable oil polyols (PUs). Researchers have tried to control the length of side chains either by bio-based polyester diols,^24^ or by rosin-based chain extender^30^ but found not ideal for biological applications due to their shape recovery below body temperature. PU based polyurethane SMPs had ability to recover its shape to maximum degree but different chain extenders and catalyst were applied to control their elastic properties^25,31,32^ as shown in Table 6. Considering these limitations, ecofriendly SMPUs, with recyclable nature but high mechanical strength, have always been in dire need.

In this research, modified sesame oil-based polyols have been utilized to formulate SMPUs, without any chain extender or catalyst, but with quick shape recovery at very low thermal actuation. Two step treatment *i.e.* epoxidation and hydroxylation, has been performed to obtain suitable polyol as plasticizer for shape memory property.

## 2 Materials and method

### 2.1 Materials

Sesame seed oil (SSO) extracted from sesame seed at commercial extracting unit, hydrogen peroxide (35%, Sigma-Aldrich), formic acid (DAEJUNG Chemicals & Metals), glacial acetic acid (100% pure, Merck KGaA), orcein reagent (Sigma-Aldrich), pure ethyl alcohol (Sigma-Aldrich), toluene 2,4-diisocyanate (TDI, extra pure, DAEJUNG Chemicals & Metals), benzene (Sigma-Aldrich), paraffin oil (Sigma-Aldrich) were utilized as raw materials in this practical work.

### 2.2 Method

#### 2.2.1 Epoxidation of SSO (ESSO)

A mixture of acetic acid (0.73 moles) and 35% hydrogen peroxide (2.35 moles) was refluxed at 40 °C for 15 minutes, SSO (0.08 moles) was added dropwise while stirring and refluxing (40 °C). After complete addition of SSO, refluxing was continued for 2.5 hours at 40 °C and 30 minutes at 130 °C. Then reaction mixture was allowed to cool at room temperature. The upper layer with bright yellow colour was separated and placed in desiccator for 6 hours. The final product (ESSO) was obtained with 90.22% yield.

#### 2.2.2 Hydroxylation of ESSO (HSSO)

A mixture of ethanol (0.804 moles), deionized water (1.66 moles) and 30 ml organic acids (acetic acid : formic acid by vol. 1 : 1) was heated at 65 °C with vigorous stirring (1500 rpm). ESSO (0.022 moles) was added dropwise and refluxed, while stirring, for 30 minutes at 65 °C. Then reaction mixture was allowed to cool at r.t. HSSO layer (lower layer) was separated from aqueous layer and placed in desiccator for 6 hours. 90% yield of HSSO (pale-yellow color) was obtained.

#### 2.2.3 Preparation of HSSO-PU

**Scheme 1:**
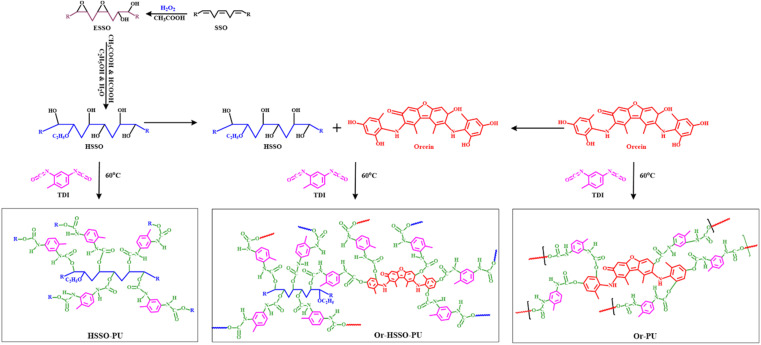
Synthesis of HSSO-PU, Or–HSSO-PU and Or-PU.

HSSO (0.0074 moles) was heated at 60 °C, while stirring (650 rpm), for 5 minutes, and TDI (0.028 moles) was added dropwise while vigorously stirring at 60 °C to avoid bubbling. The mixture was heated for 10 minutes while vigorously stirring. Viscous dispersion was then shifted into mold and set to dry for 72 hours at room temperature. Very light-yellow thin layer of HSSO-based PU about 1 mm thickness was obtained. Proposed reaction scheme is presented in Scheme 1.

#### 2.2.4 Preparation of Or-PU

Orcein (0.0004 moles) was dissolved with stirring in ethanol (0.338 moles) at r.t. and heated at 60 °C for 5 min. Then TDI (0.0417 moles) was added dropwise in 2 min and heated while stirring at 60 °C for 10 minutes. The viscous dark red material was shifted into plastic mold and allowed to cure for 72 hours. Or-PU with 1 mm thickness was obtained. Proposed reaction scheme is presented in Scheme 1.

#### 2.2.5 Preparation of Or–HSSO-PU

Orcein (0.0004 moles) ethanol (0.338 moles) solution was mixed with HSSO (0.00165 moles) and stirred for 5 min. Then TDI (0.0556 moles) was added dropwise (in 3 minutes) with stirring and heating at 60 °C for 10 minutes. The dark reddish viscous liquid was shifted into mold and set to dry for 72 hours. Or–HSSO-PU with thickness of 1 mm was obtained. Proposed reaction scheme is presented in Scheme 1.

## 3 Characterizations

Synthesized PU samples have been analyzed through FTIR-(IRSpirit) Shimadzu with diamond ATR in range 500–4000 cm^−1^, TGA/DSC (STA SKZ1060A Industrial Co., Limited) from room temperature to 500 °C at ramp rate of 10 °C min^−1^ for 5 mg sample in Al crucible using oxidative environment and air as purge gas, light microscope (IRMECO GmbH & Co., IM-910). The morphology and surface roughness of synthesized PU composites have been studied by scanning electron microscope (SEM, ZEISS EVO, Carl Zeiss) and atomic force microscopy (AFM). Molar mass of treated SSO was determined by ebullioscopic method (eqn (1)) taking benzene as solvent.

1 Δ*T*_b_ = *K*_b_*m*

where Δ*T*_b_ = elevation in boiling point of specific solvent, *K*_b_ = ebullioscopic boiling constant of specific solvent and *m* = molality of unknown sample. For benzene, the value of ebullioscopic boiling constant is 2.53 °C kg mol^−1^.^33^

Iodine value (IV) was determined by reported method.^34^ 0.2 g sample with 10 ml chloroform was stirred with 30 ml of Hanus solution for 15 minutes. 10 ml of 15% KI and 100 ml DI water were added and titrated against 0.1 N Na_2_S_2_O_3_ till yellow colour, added 2–3 drops of starch indicator and titrated again till blue colour. Calculated IV by using eqn (2).2
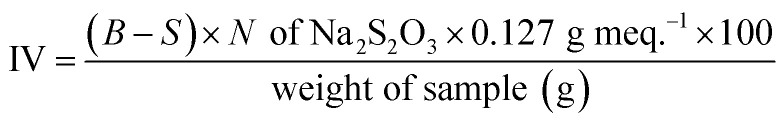
where *B* and *S* are the volume used of Na_2_S_2_O_3_ against blank solution and sample and *N* is the normality of Na_2_S_2_O_3_.

Epoxy value (EV) was determined by HCl–acetone titration method.^35^ 0.25 g sample was stirred in 5 ml HCl (0.1 N) and 35 ml of acetone. 5 ml mixture, with 2–3 drops of indicator, was titrated against 0.1 N NaOH till pink color. EV value was calculated by eqn (3).3
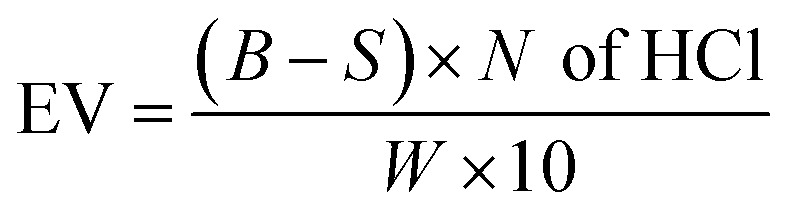
where *S* and *B* are the volume used of NaOH against sample and blank solution, and *W* is the weight of sample.

Samples' density was determined by using mass/volume relationship.^36^ Gel contents were measured by a method reported in literature.^37^ Calculated amount of PU samples (HSSO-PU, Or–HSSO-PU and Or-PU) was soaked in 20 ml of dichloromethane (DCM) for 24 hours. Then samples were removed from solvent and dried at 40 °C, and weighted to calculate gel contents by using eqn (4) and (5).4
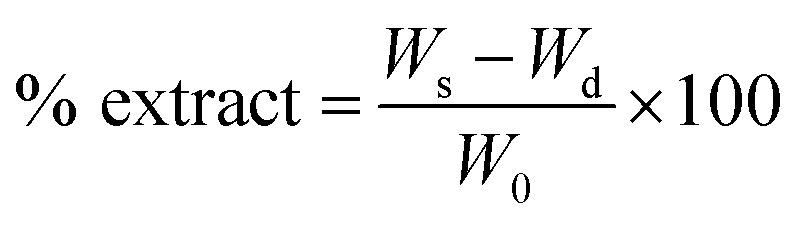

5 % gel content = 100 − % extract

where *W*_s_ is weight of sample and *W*_d_ is weight of dried sample.

Water absorption capacity was determined by a method reported in literature.^38^ Weighed PU samples were dipped in deionized water for 48 hours. At regular time intervals samples were removed form water, dried and weighed to determine water absorption by eqn (6).^15^6
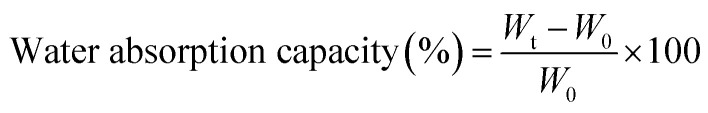
where *W*_t_ = weight of sample after dipping, *W*_0_ = weight of dried sample.

Hemolytic activity, antioxidant activity and antibacterial activity of synthesized PU composites have been determined by reported methods^39–41^ using eqn (7) and (8), respectively.7
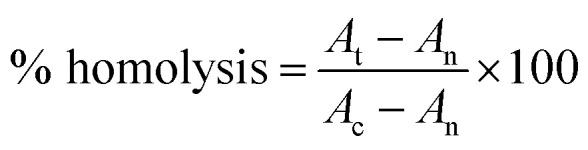
where *A*_t_ is the absorbance of the test sample. *A*_n_ is the absorbance of the control (saline control) *A*_c_ is the absorbance of the control (Triton control).8
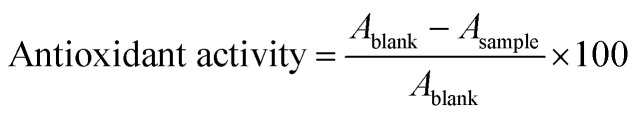
where; *A*_blank_ is the absorbance of the control reaction (containing all reagents except the extract) and *A*_sample_ is the absorbance of the mixture containing the extract.

### 3.1 Shape memory test

**Fig. 1:**
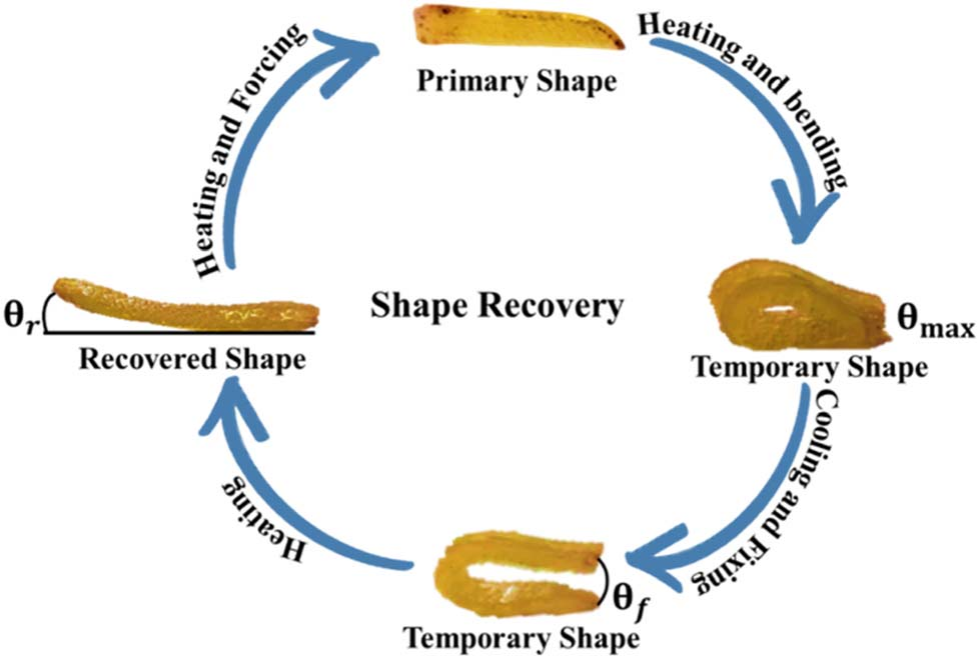
Shape memory bending test.

Rectangular strips of PU films having dimension 3 cm × 6 mm × 3 mm (Fig. 1) were used to study shape memory behavior (Fig. 1).

For shape memory study, films were heated at 54 °C, bent and cooled to maintain temporary shape. Thermal stimulus (50–60 °C) was provided to films for restoration of original shape. The displacement of shape from *θ*_max_ while cooling and fixing is known as angle of fixity (*θ*_f_). Attained position by bent film while displacing towards original shape after heating is known as recovered shape and the difference of angle from starting position is known as angle of recovery (*θ*_r_). Cycles of conversion original → temporary → original shape were repeated five times. Ability of PU films to gain temporary shape and restoration to original shape under the influence of temperature is the shape recovery rate (*R*_r_) and shape fixity rate (*R*_f_), calculated with eqn (9) and (10).^15^9
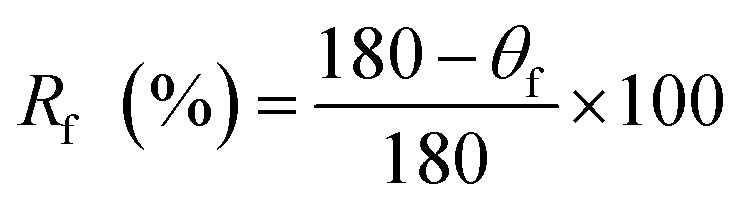
10
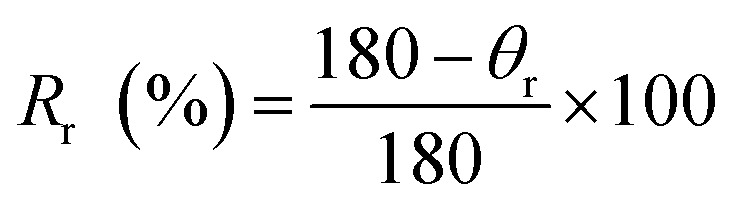


## 4 Results and discussions

### 4.1 Physico-chemical analyses

Molecular mass (MM), iodine value (IV), epoxy value (EV) and density of unmodified and modified SSO were calculated to confirm the effectiveness of modification protocol. Upsurge in MM and density of modified samples in comparison to unmodified sample, confirmed the change in molecular assembly. IV difference signposted the effective consumption of unsaturated contents during two step modification. EV counter-confirmed the effectiveness of two-step modification protocol (*i.e.* epoxidation and hydroxylation) (Table 1).

**Table 1:** Characteristic data of samples

Sample ID	MM (g mol^−1^)	IV	EV	Density (g cm^−3^)
SSO	1103.8	129.19	104.76	0.9130
ESSO	1290.3	114.20	131.10	0.9523
HSSO	1322.6	100.66	104.90	1.0889

### 4.2 FTIR analyses

**Fig. 2:**
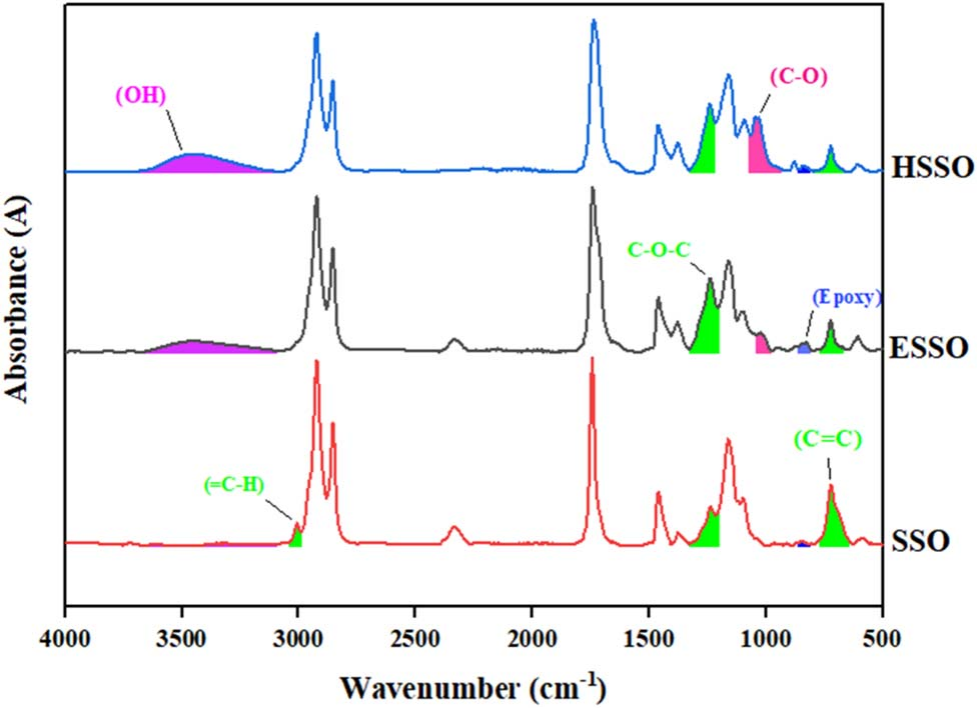
FTIR of SSO, ESSO and HSSO.

SSO to ESSO modification was studied by FTIR data (Fig. 2). SSO spectra showed peaks of different functional groups (*e.g.* C=C, C–O–C and =C–H) at 723 cm^−1^, 1242 cm^−1^ and 3008 cm^−1^ respectively.^42,43^ After treatment, ESSO spectra showed a reduction in C=C peak with peak area condensed thrice, whereas, absorbance peak of C–O–C became sharp and wide with peak area almost doubled. At the same time, epoxy peak with peak area 0.62 was observed at 826 cm^−1^ in ESSO spectral line.^42^ Disappearance of =C–H peak (3008 cm^−1^)^43^ in ESSO, may be attributed to conversion of alkene into epoxy, confirmed by the appearance of epoxy peak in ESSO. The peaks present at 2347 cm^−1^ and 1738 cm^−1^ represent carbonyl functional group.^43^ FTIR spectra of HSSO (Fig. 2) obtained after treating ESSO with organic acids (acetic acid and formic acid) in ethanol and water, showed a reduction in C–O–C peak at 1242 cm^−1^ and also in epoxy peak at 826 cm^−1^. Moreover, –OH peak appeared at 3502 cm^−1^.^43^ The conformation of OH induction may be done with C–O peak formation at 1037 cm^−1^.^43^

**Fig. 3:**
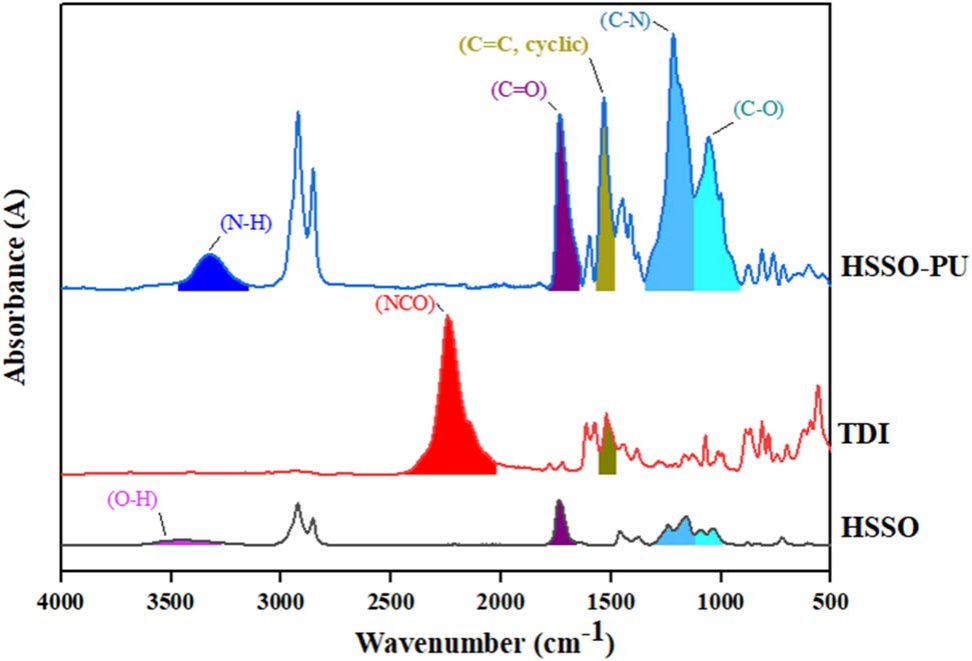
FTIR of HSSO-PU and its reactants.

HSSO, as biobased diol, upon reaction with TDI resulted in HSSO-PU (Fig. 3). Peaks at 3502 cm^−1^ and 2245 cm^−1^ in HSSO FTIR spectra, represent OH in HSSO and NCO in TDI, respectively.^33^ Upon reaction of HSSO with TDI, disappearance of both OH and NCO peaks in product (Fig. 3) confirmed the consumption of these functional groups along with the formation of a new functional group C–O at 1054 cm^−1^.^43^ Breakdown of C=N bond in TDI, resulted in new bonds, *i.e.* C–N with peak at 1219 cm^−1^,^33^ N–H with peak at 3333 cm^−1^,^33^ and carbonyl with peak at 1738 cm^−1^.^43^ Peak at 1533 cm^−1^ indicated the presence of cyclic alkenes.^33^

**Fig. 4:**
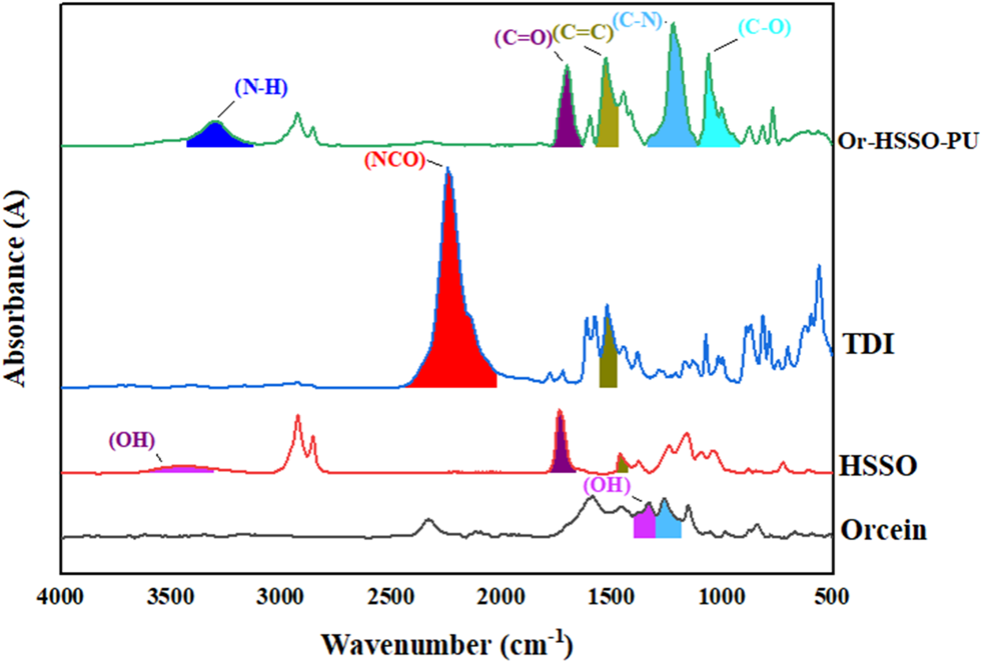
FTIR of Or–HSSO-PU and its reactants.

Or–HSSO-PU sample along with ingredients was analyzed through FTIR (Fig. 4). Peaks of NCO and OH were found at 2245 cm^−1^ and 1328 cm^−1^, respectively,^15,33^ while HSSO hydroxyl group was observed at 3502 cm^−1^.^33^ After reaction of orcein and HSSO with TDI, both NCO and OH peaks disappeared rising new peaks in product. It is assumed that polyols OH reacted with C=N in TDI, resulting in CO bond between TDI and polyol with peak at 1054 cm^−1^.^43^ Similarly, N linked with H and peak appeared at 3299 cm^−1^.^15^ Likewise, breakup of double bond between C and N resulted in single bond left with peak at 1219 cm^−1^.^15,33,43^ Carbonyl peak at 1703 cm^−1^, also increased in size. The peak of cyclic C=C appeared at 1533 cm^−1^.

**Fig. 5:**
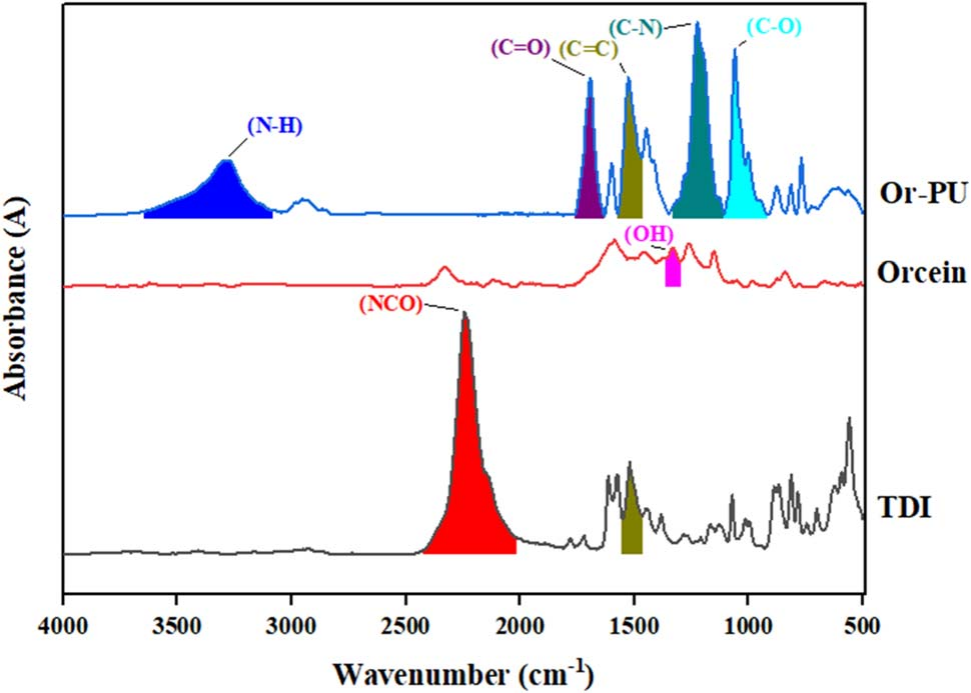
FTIR analysis of Or-PU and its reactants.

Similarly, FTIR analysis was applied on Or-PU and its reactants to observe different functional group formed (Fig. 5). Phenolic OH group was observed at 1328 cm^−1^. After reaction between polyol and isocyanate, NCO and OH functionalities disappeared with new group formation in product, *i.e.* N–C, C–O and N–H. Peaks of NCO and OH were at 2254 cm^−1^ and 1328 cm^−1^ respectively.^15,33,43^ Appearance of these new peaks in Or-PU confirmed the synthesis of product as shown in Fig. 5.

### 4.3 TGA

**Fig. 6:**
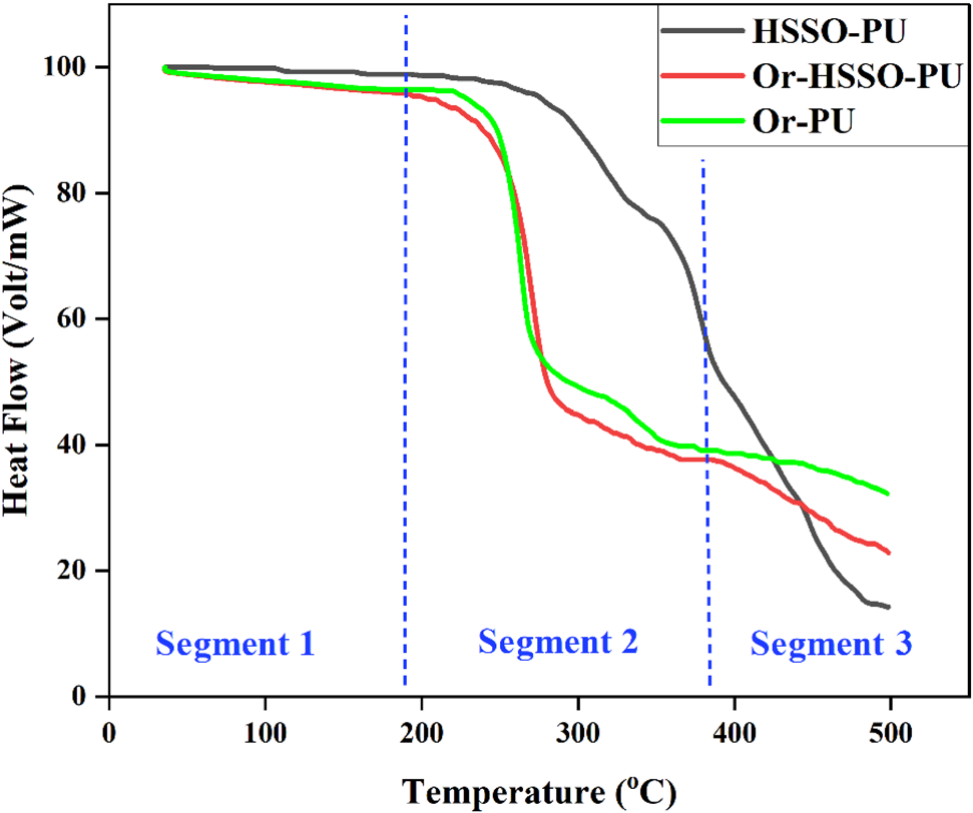
TGA of PU composites.

Thermal stability of prepared samples has been studied with TGA (Fig. 6). Data revealed that incorporation of orcein imparts thermal instability into samples, which might be attributed to higher oxidizable contents on its surface. Thermal degradation of sample prepared from biobased material was observed least in first two segments, which might be attributed to least oxidizable contents on its surface. In third segment (above 400 °C) thermal stability pattern reversed *i.e.* biobased polyol containing PU degraded quickly in comparison to samples having orcein as polyol. This reversal in degradation pattern may be attributed to fact that orcein based PU samples lost most of oxidizable components in first two segments and became thermally stable whereas biobased polyol containing PU, at 450 °C (Fig. 6), decomposed carbon chains into smaller fragments, prone to thermal degradation.^44^

### 4.4 DSC

**Fig. 7:**
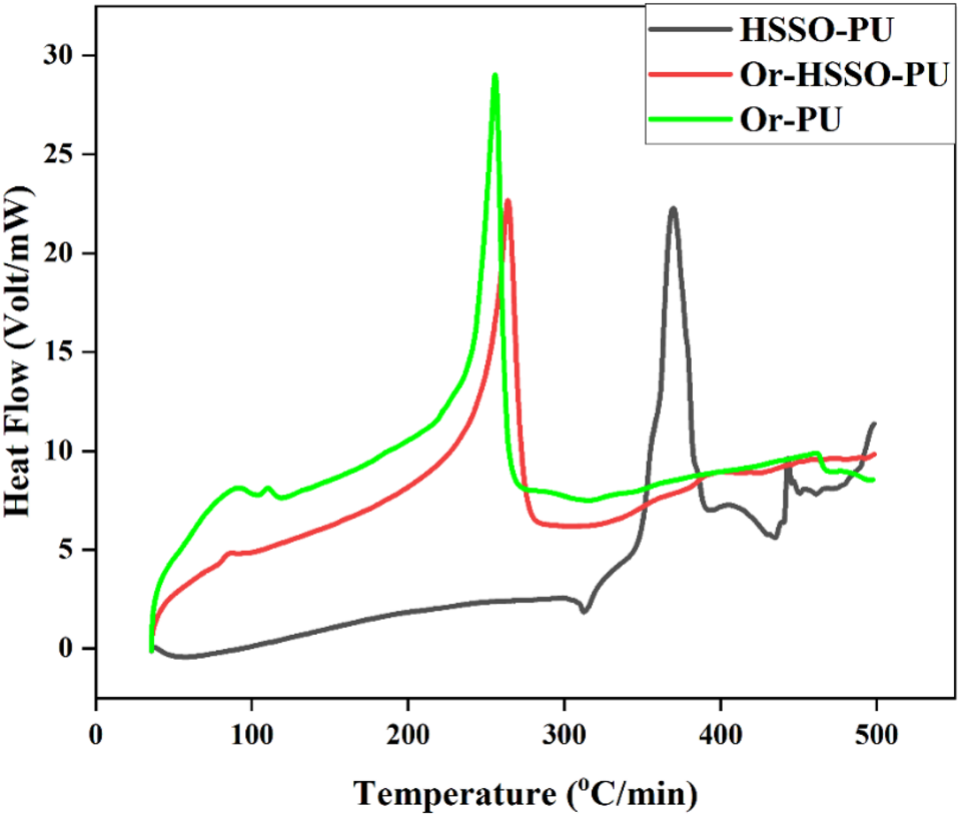
DSC of PU composites.

Prepared PU samples were analyzed through DSC (Fig. 7, Table 2). Comparative analysis, on the basis of increasing biobased polyol component, showed an augmentation trend in *T*_g_ of hard segment (TDI), signposting micro-phasic separation of hard and soft segments of PU samples by adding HSSO.^45^ About 100 °C increase in thermal melt of HSSO-PU than Or-PU is due to its well-defined microcrystalline structure which signpost increased cross linking density of rubbery soft segments (polyols), moreover 10 cal. low Δ*H*_m_ of HSSO-PU than Or-PU represents less thermal dissipation of amorphous HSSO.^46^ This data evidenced a vivid micro-phasic level separation of hard and soft segments in HSSO-PU.

**Table 2:** DSC data of prepared PU samples

Sample ID	*T*_g,HS_ (°C)	Thermal melt	
		Temperature (°C)	Δ*H*_m_ (cal.)
HSSO-PU	322.8	369.6	132.5
Or–HSSO-PU	88.6	264.4	136.4
Or-PU	90.4	255.6	141.9

Thermal melt enthalpy increases with increasing polyol content in PU structure.^45^ Or-PU showed highest Δ*H*_m_ of all samples indicating highest alcoholic content involvement in polymerization, which might be attributed to condensed structure of orcein, but high thermal dissipation capacity of orcein decreased thermal melt temperature of Or-PU than HSSO-PU.

### 4.5 Gel content & water absorption capacity

Gel content analysis was applied on prepared PU samples (Table 3). HSSO-PU was found to have the highest gel contents (99.27%) in comparison to Or-PU (98.32%) and Or–HSSO-PU (97.66%) verifying huge quantity of constituents to be crosslinked during synthesis of PU samples.

**Table 3:** Gel content (%) and water absorption capacity (%) of PU samples

Samples	Gel content (%)	Water absorption capacity (%)						
		0 h	1 h	2 h	3 h	4 h	24 h	48 h
HSSO-PU	99.27	0	3.9	4.09	4.09	9.42	9.71	14.94
Or–HSSO-PU	97.66	0	0.47	1.42	4.42	3.63^a^	7.4^a^	5.53^a^
Or-PU	98.32	0	3.14	5.45	6.7	7.54	10.06	15.01

a Dissolution was observed for a small portion of sample.

Water absorption capacity of prepared PU samples was also studied to confirm their hydrophilic nature and biodegradation behavior. Water absorption results (Table 3) indicated an upsurge with temperature rise, which links soft segment's interaction with water. Swelling of material relates inversely with crosslinking density of the constituents, *i.e.* Or-PU composite had high swelling proportion as compared to HSSO-PU composite due to low crosslinking density.

### 4.6 Morphology

**Fig. 8:**
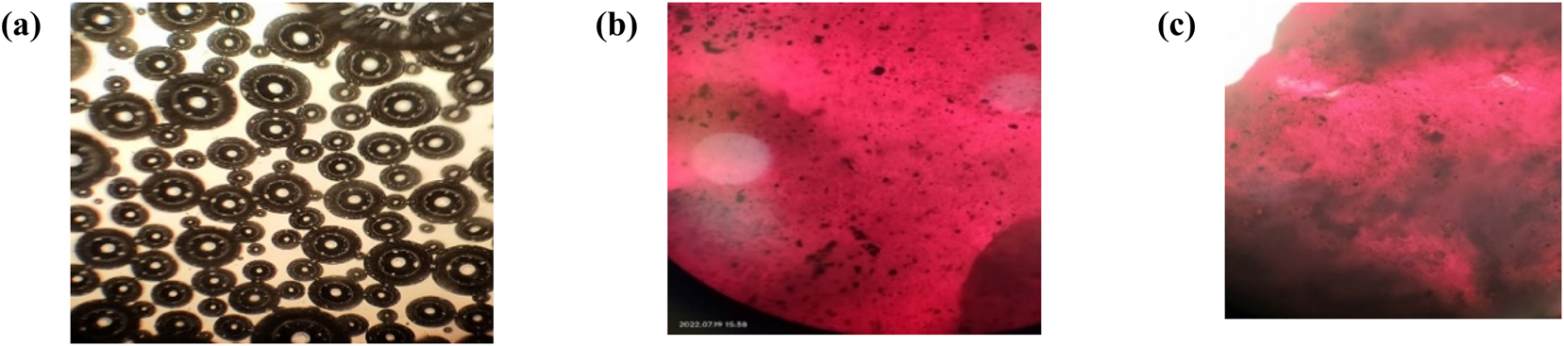
Microscopic images of (a) HSSO-PU (b) Or–HSSO-PU (c) Or-PU.

Microscopic images of HSSO-PU, Or–HSSO-PU and Or-PU (Fig. 8) presented segmental separation of soft and hard components in HSSO-PU, whereas Or-PU showed a homogeneous dispersion of orcein throughout PU sample. Segmental separation type configuration in HSSO-PU has been supported by DSC thermal melt data, which specified well-defined microcrystalline hard segments.

### 4.7 SEM and AFM

**Fig. 9:**
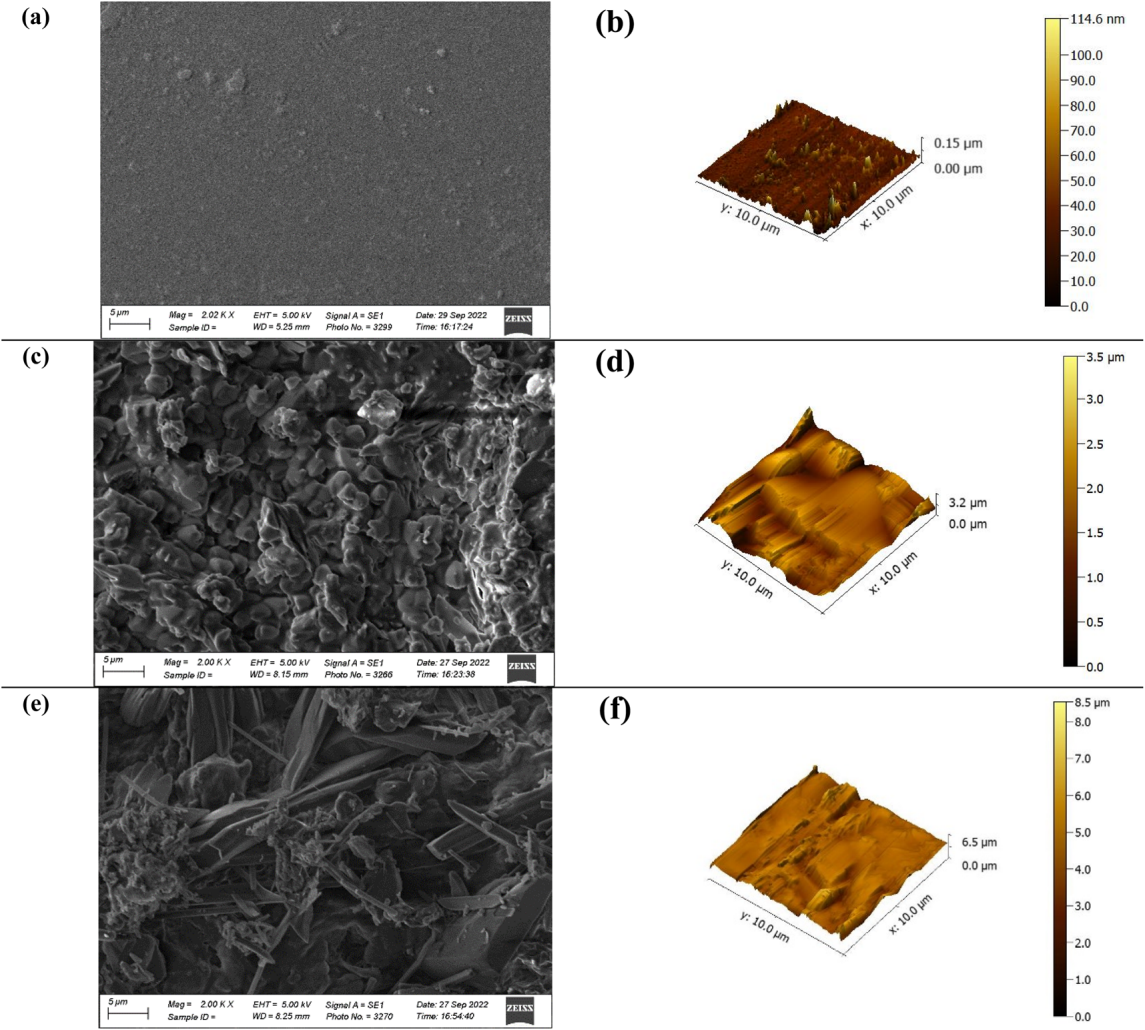
SEM and AFM diagrams of (a and b) HSSO-PU, (c and d) Or–HSSO-PU, (e and f) Or-PU.

SEM and AFM analyses of prepared samples (Fig. 9) at μ level revealed homogeneity and smoothness in sample with biobased plasticizer (HSSO). On the contrary, orcein amalgamation imparted non-homogeneity and surface roughness (almost 40 folds to biobased plasticizer), which might be considered for the structural damage during shape memory studies.

### 4.8 Bioactivities

Hemolytic activity showed mild toxicity at 12 mg ml^−1^ concentration, which shrank with concentration drop (Table 4). The highest was shown by Or-PU at 14.2 ± 0.14 while the lowest was by HSSO-PU at 12.3 ± 0.17 at 12 mg ml^−1^ concentration. Likewise, antioxidant potential of HSSO-PU has been observed at a high antioxidant value of 75.34 ± 0.79 followed by Or–HSSO-PU and Or-PU that is 74.5 ± 0.77 and 69.22 ± 0.69, respectively.

**Table 4:** Toxicity of PU composites at different concentrations

Concentration (mg ml^−1^)	Toxicity				Antioxidant activity			
	HSSO-PU	Or–HSSO-PU	Or-PU	Triton X-100	HSSO-PU	Or–HSSO-PU	Or-PU	Ascorbic acid
12	12.3 ± 0.17	13.2 ± 0.11	14.2 ± 0.14	97.5 ± 1.8	75.34 ± 0.79	74.5 ± 0.77	69.22 ± 0.69	95.93 ± 1.5
6	10 ± 0.1	10.8 ± 0.09	11.4 ± 0.09		71.72 ± 0.7	70.1 ± 0.71	66.43 ± 0.58	90.62 ± 1.1
3	7.9 ± 0.08	8.7 ± 0.06	9.1 ± 0.07		63.09 ± 0.61	68.4 ± 0.66	55.57 ± 0.51	88.71 ± 0.99
1.5	3.8 ± 0.05	4.6 ± 0.06	7.8 ± 0.04		55.84 ± 0.48	65.6 ± 0.55	54.03 ± 0.43	85.22 ± 0.83
0.75	1.8 ± 0.03	2.8 ± 0.02	3.7 ± 0.03		53.06 ± 0.4	63.3 ± 0.51	46.23 ± 0.33	80.29 ± 0.78

### 4.9 Antibacterial activity

Antibacterial activity of samples showed that *E. coli* is sensitive bacteria against samples that show maximum zone of inhibition in HSSO-PU and Or-PU, while *B. subtilis* and *S. aureus* also show a zone of inhibition against HSSO-PU and Or–HSSO-PU. The antibacterial activity of HSSO-PU, Or–HSSO-PU, and Or-PU at sample concentration of 12 mg ml^−1^ was observed to be 1.8, 1.1, 1.1 for *E. coli*, 2.8, 1.7 and 1.7 for *B. subtilis* and 1.9, 2.4 and 1 for *S. aureus*, respectively. For 6 mg ml^−1^ sample concentration, the antibacterial activities of HSSO-PU, Or–HSSO-PU, and Or-PU were 1.1, 0 and 0.5 for *E. coli*, 2, 0 and 1.2 for *B. subtilis*, and 1.2, 0 and 0 for *S. aureus*, respectively. While HSSO-PU exhibits bacterial resistance behaviour with 0.7, 0.5, and 0.3 antibacterial activity for *E. coli* for lower sample concentrations (3, 2.5, and 0.75 mg ml^−1^). However, other samples with lower sample concentrations failed to exhibit antibacterial efficacy against referred bacterial species.

### 4.10 Shape memory

**Fig. 10:**
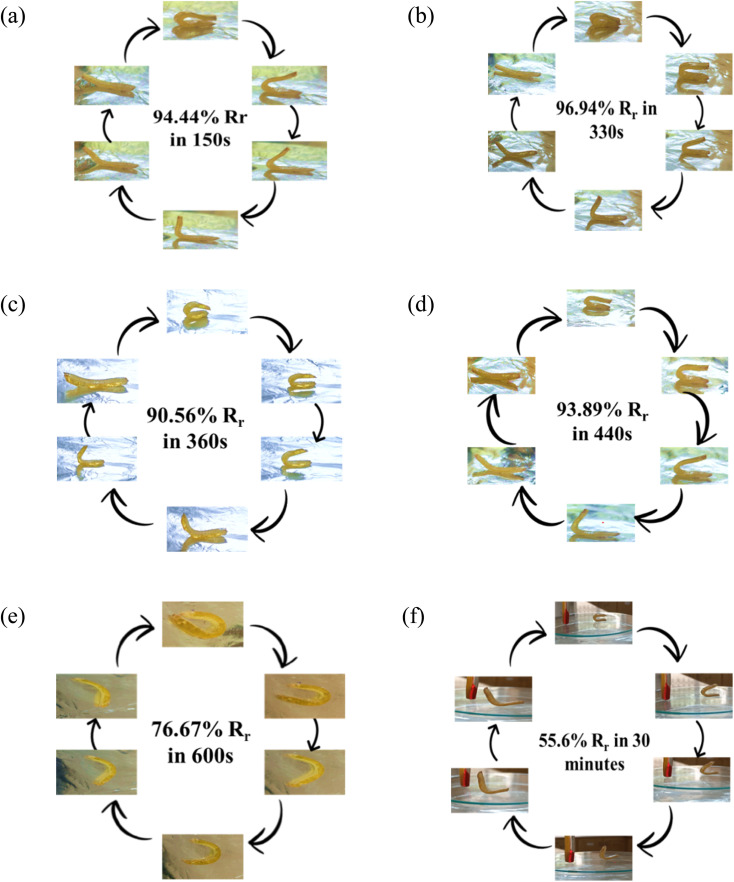
Shape memory study of HSSO-PU at (a) 60 °C, (b) 55 °C, (c) 54 °C, (d) 52 °C, (e) 51 °C, (f) 40 °C.

**Fig. 11:**
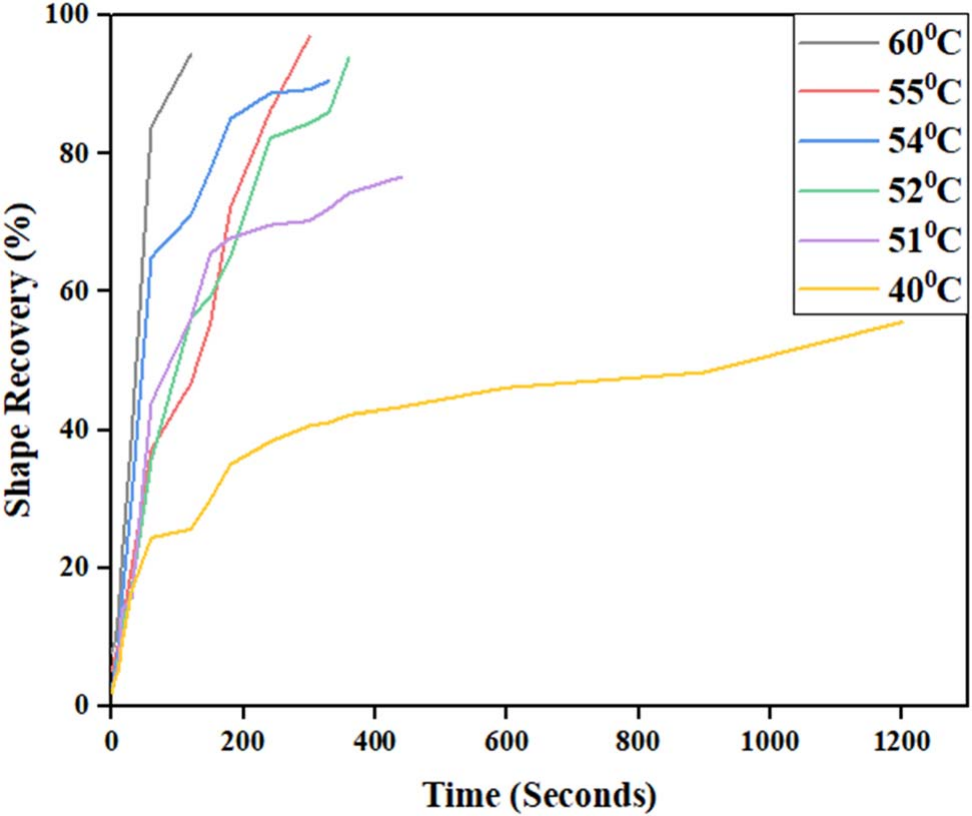
Shape recovery graph of HSSO-PU composite at different temperatures.

Shape memory of PU samples (HSSO-PU, Or–HSSO-PU and Or-PU) was studied under different temperatures (Fig. 10). Or–HSSO-PU and Or-PU showed rigidity till 40 °C and broke but above 40 °C started melting and did not show any shape recovery behavior. This may be attributed to strong thermal dissipation behavior of orcein, which caused melting of sample at a specific temperature through structural damage. On the other side, HSSO-PU started showing flexibility at 50 °C. Shape memory and shape fixing behaviors of HSSO-PU films were studied in temperature range of 50–60 °C (Table 5). Fast shape recovery was found with temperature rise but with more shape fixing, which may be attributed to structural damages of HSSO-PU at 60 °C (Fig. 11).

**Table 5:** Shape recovery behavior of HSSO-PU at different temperatures

Sample ID	Temperature (°C)	Recovery time (s)	Shape recovery rate% (*R*_r_)	Ref.
HSSO-PU	60	150	94.44	This work
	55	330	96.94	
	54	360	90.56	
	52	440	93.89	
	51	600	76.67	
	40	30 min	55.6	
SMPUs	35–45	NR	98	47
PU/PEEAMA	60	5	96	48

**Table 6:** Composition and *R*_r_ time comparison of body temperature sensitive PU

Sr. no.	Polyol	Isocyanate	Catalyst	Chain extender	*R*_r_ time (seconds)	Ref.
1	Co-PLAols	IPDI	SnOct_2_	BDO, HDO, EG	60	25
2	PCL diol	MDI-50	SnOct_2_	N/R	15	31
3	Co-PLA tetraol	HDI	SnOct_2_	PETP	101	32
4	HSSO	TDI	N/A	N/A	30 min	This work

**Fig. 12:**
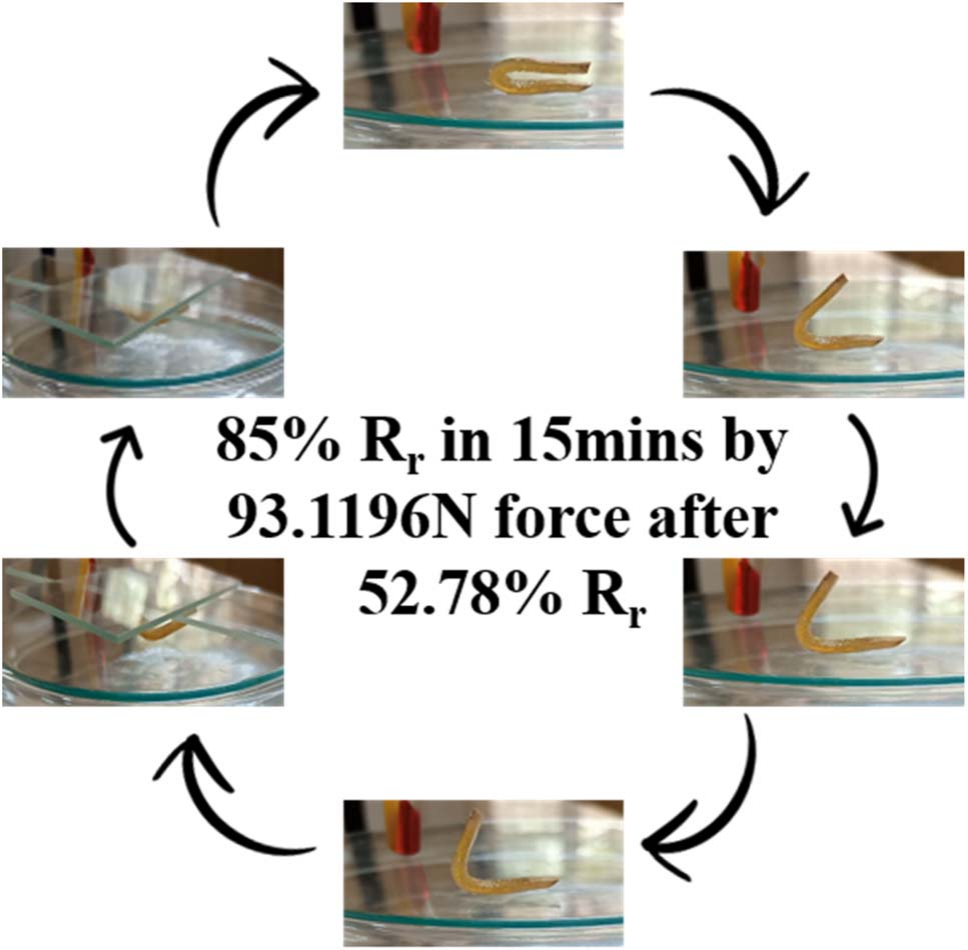
Shape memory study of HSSO-PU at 36 °C.

Biodegradability and water uptake response of PU samples containing HSSO (Table 3) has widened the application scope of these materials in textile as well as biomedical not only as drug carrier but also as shape recovery materials. To recognize shape memory response of HSSO-PU at human body temperature, shape recovery at 36 °C was studied (Fig. 12). 52.78% shape recovery was found without any force whereas 85% shape recovery was observed at 93.1196 N force.

Upon comparison of shape recovery data from already published works, it is being claimed that current research has neither used any types of chain extenders nor catalyst to support shape recovery rate at body temperature, furthermore, not any specially designed commercial product to impart flexibility (MDI-50) has been incorporated in molecular design. This outcome *i.e.* synthesis without special chemical, will support HSSO-PU's expanded biomedical application domain.

## 5 Conclusion

In this research biobased polyols have been designed through an eco-friendly and facile approach. SSO has been treated to obtain hydroxylated plasticizer (HSSO), which has induced low thermal actuation shape recovery with high thermal stability in PU samples, whereas orcein has imparted rigidity and easy thermal degradation. HSSO has framed separate and well-defined hard and soft segments in PU. Prepared samples have 100% thermal stability till 270 °C, but shape recovery response initiated at very low thermal stimulus *i.e.* 36 °C, which has made it a suitable candidate for self-tightening structures not only in textiles and biomedical applications like dental implants, but also in all applications that require shape recovery at very low temperature.

## Conflicts of interest

There are no conflicts to declare.

## Supplementary Material
